# Determination of ochratoxin A in pig tissues using enzymatic digestion coupled with high-performance liquid chromatography with a fluorescence detector

**DOI:** 10.1016/j.mex.2016.03.006

**Published:** 2016-03-10

**Authors:** Luci Giacomo, Vanni Michele, Ferruzzi Guido, Mani Danilo, Intorre Luigi, Meucci Valentina

**Affiliations:** Department of Veterinary Science, University of Pisa, Italy

**Keywords:** Enzyme assisted digestion coupled to HPLC-FLD, Enzyme assisted digestion, Ochratoxin A, Pig, Muscle, Kidney, Liver

## Abstract

We present a new method for the rapid analysis of ochratoxin A (OTA) in pig tissues (muscle, liver and kidney) using enzymatic digestion (ED) coupled to high-performance liquid chromatography with a fluorescence detector (HPLC-FLD). OTA was digested with a 1% pancreatin solution in a phosphate buffer and then cleaned with ethylacetate. After being evaporated to dryness and re-dissolved, the sample was determined using HPLC-FLD. The method was validated taking into account the currently permitted limit of 1 μg/kg OTA in pork meat and derived products in Italy. The recovery was higher than 90%. Intra- and inter-day repeatability expressed as RSD were less than 7%. The LOD and LOQ were 0.001 and 0.002 μg/kg, respectively. Our method is more efficient, easier, and cheaper than conventional clean-up procedures (liquid–liquid extraction).

• The aim of the study was to develop and validate a quantitative HPLC-FLD method based on ED followed by a chromatographic analysis without any previous clean-up or concentration step for the detection of OTA in pig tissues.
• The ED method showed a 90%+ recovery, and intra- and inter-day RSD less than 7%.
• This method is simple, rapid, easy to use, and consumes low amounts of organic solvents.

## Graphical abstract

**Figure:**
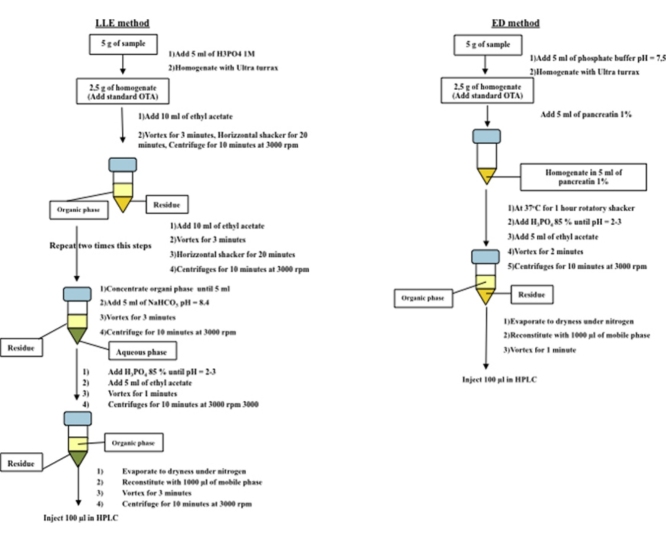

## Method details

Ochratoxin A (OTA) is a secondary toxic metabolite of various *Penicillium* and *Aspergillus* fungi, which is widely distributed in cereals [1]. OTA is nephrotoxic and immunotoxic. IARC classified OTA as a possible human carcinogen (Group 2B) [2]. Long-term exposure to OTA in humans has been implicated in Balkan endemic nephropathy (BEN) and is associated with urinary tract tumors because of the high OTA levels detected in food samples and in blood or urine from affected patients. As cereals are widely used in animal feed, animals are continuously exposed to OTA through the consumption of contaminated feed, which can lead to the accumulation of this mycotoxin in meat and meat products [3].

Some countries have set maximum levels of OTA in meat or animal products, such as Denmark (pig kidney 10 μg/kg, pig blood 25 μg/ml), Romania (pig kidney, liver, and meat 5 μg/kg), and Italy (pig derived products 1 μg/kg) [4]. As one of the main sources of meat for humans, it is essential to focus on the residues of OTA in pork. Given that mycotoxins have a particularly complex matrix, it is more difficult to determine them in meat than in cereal grains. The most common methods for the determination of OTA in animal tissues are performed by extraction with chloroform, followed by a clean-up with immunoaffinity columns or liquid–liquid partitioning [5], [6], [7]. However, conventional procedures need a large amount of organic solvents, which are environmentally harmful and hazardous to humans. The aim of the present study was to develop and validate a new enzymatic digestion method coupled with HPLC-FLD for OTA quantitative determination in pig tissues.

## HPLC-FLD analysis

The chromatographic system consisted of a Jasco 880 pump and a Jasco 821 fluorescence detector (Jasco, Tokyo, Japan). JascoBorwin software was used for data processing. The excitation wavelength (λ_ex_) and emission wavelength (λ_em_) were set at 380 and 420 nm, respectively. The reversed-phase column was a HAISIL HL, C_18_, 5 μm, 150 mm × 4.6 mm (Higgins Analytical, USA). The column was kept at room temperature. The HPLC was operated with a mobile phase system consisting of a methanol-phosphate buffer solution pH 7.5 (0.03 M Na_2_HPO_4_, 0.007 M NaH_2_PO_4_) 50/50% v/v at flow rate of 1 ml/min.

OTA (from *Aspergillus ochraceus*) (M 403.8) reference standard was purchased from Sigma (Milan, Italy). The OTA standard was dissolved in a toluene-acetic acid mixture (99:1%, v/v) to give a stock solution of 200 μg/ml, which was stored at −20 °C until use. Working solutions were prepared by diluting the stock solution with the mobile phase consisting of a methanol-sodium phosphate buffer (pH 7.5) 50:50% v/v. HPLC-grade water, methanol, ethylacetate and acetonitrile were purchased from VWR (Milan, Italy). The pancreatin enzyme (from porcine pancreas) was purchased from Sigma (code P1750, Milan, Italy), and was stored at −20 °C until use.

## Standard liquid–liquid extraction (LLE)

OTA was extracted according to Meucci et al. [8] with slight modifications. A 5 g liver, kidney or muscle sample aliquot was homogenized with 5 ml of phosphoric acid 1 M using an Ultra Turrax T25 homogenizer for a few minutes. A 2.5 g aliquot of the homogenate was transferred into a centrifuge tube, extracted with 10 ml of ethylacetate, vortexed for 3 min, shaken for 20 min on a horizontal shaker, and then centrifuged for 10 min at 3000 rpm. The organic phase was removed, the residue re-extracted, as above, and the organic phases combined. The volume of the organic phase was reduced to approximately 5 ml and back-extracted with 5 ml of NaHCO_3_ pH 8.4, vortexed for 1 min, and centrifuged for 10 min at 3000 rpm. The aqueous extract was acidified to pH 2.5 with H3PO4 85% and briefly sonicated to strip the CO_2_ formed. OTA was finally back-extracted into 5 ml ethylacetate, vortexed for 1 min, and centrifuged for 10 min at 3000 rpm. The organic phase was evaporated to dryness under nitrogen stream, reconstituted in 1000 μl of mobile phase, and a 100 μl aliquot injected into HPLC.

## Enzymatic digestion method (ED)

OTA is a weak acid (pKa 4.4 and 7.3 for the carboxyl and the hydroxyl group, respectively) and can be extracted from a water phase into a less polar solvent only at pH < 7, as under neutral and alkaline conditions it is present in the dissociated form. In most studies, OTA has been extracted from animal tissues by chloroform after acidification with a solution of hydrochloric or phosphoric acid [7]. OTA has been determined in kidneys by enzymatic extraction in two old methods using subtilisin A or papain prior to the extraction [9], [10]. More recently, a method was proposed based on an enzyme-assisted extraction with pancreatin prior to purification through immunoaffinity columns for OTA in ham samples [11].

Because of the complexity of the published methods and the use of chlorinated solvents for the extraction in the vast majority of such methods, we developed a new enzymatic digestion method without immunoaffinity purification. The study was aimed at reducing the number of individual steps, while still detecting OTA in pig tissues with low levels of concentration. We decided to use pancreatin as a proteolytic enzyme because it is active in neutral medium (pH 6–8). On the other hand, enzymes such as pepsin which are active in acid medium (pH 1.5–2.5), are not suitable for OTA, because the toxin is destroyed very quickly owing to the hydrolysis of the amide bond.

Five grams of muscle, liver, or kidney sample aliquot were homogenized with 5 ml of a phosphate buffer (sodium phosphate monobasic dihydrate 0.2 M and sodium phosphate dibasic 0.2 M 20:80% v/v pH 7.5) using an Ultra Turrax T25 homogenizer for a few minutes. A 2.5 g aliquot of the homogenate was transferred into a tube and incubated at 37 °C with a solution of 1% pancreatin in a phosphate buffer (sodium phosphate monobasic dihydrate 0.2 M and sodium phosphate dibasic 0.2 M 20:80% v/v pH 7.55). Several parameters were varied in the enzymatic digestion in order to obtain the best recovery of OTA from the relevant matrix.

Different volumes of pancreatin solution were evaluated (20, 10 and 5 ml), for different incubation times (1, 2 and 3 h). The incubation was performed at 37 °C in a rotatory shaker, after which step samples were acidified with H_3_PO_4_ 85% until pH 2–3. These samples were then extracted with the same volume of ethylacetate, vortexed for 1 min, and centrifuged for 10 min at 3000 rpm. The organic phase was evaporated to dryness under nitrogen stream, reconstituted in 1000 μl mobile phase, and a 100 μl aliquot was injected into HPLC. Table 1 shows the best conditions of OTA extraction using enzymatic digestion: 5 ml of pancreatin 1% solution for 1 h at 37 °C. Experiments were performed on muscle, liver and kidney pig samples spiked with 1 ppb of OTA. Spiking solutions of OTA were prepared daily by dilution with HPLC mobile phase. For the pig muscle, liver and kidney samples, after thoroughly mixing for 30 min, the OTA-fortified homogenate was left for at least 2 h at room temperature for equilibration, and then used to assay the cleaning procedures prior to HPLC-FLD analysis. The whole analysis, including sample preparation, can be carried out in one and a half hours.

## Comparison between LLE and ED methods

**Fig. 1:**
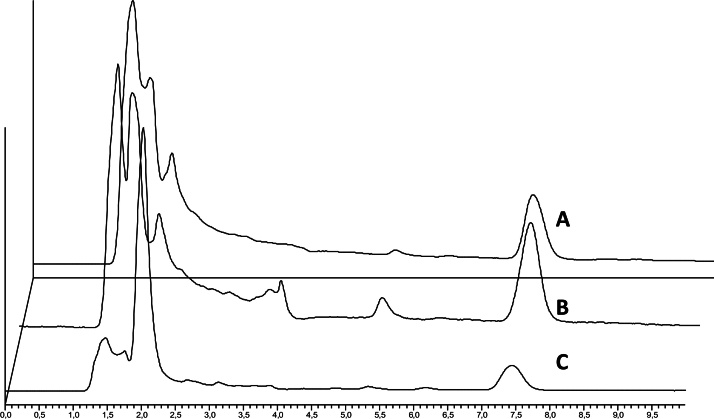
Chromatograms of a naturally-contaminated pig (A) muscle, (B) liver and (C) kidney sample extracted with ED.

We compared our ED procedure and LLE protocol in terms of their performance regarding OTA quantitative determination. The LLE protocol is conventionally used for OTA extraction from animal tissues followed by immunoaffinity or liquid–liquid partition with a sodium bicarbonate aqueous solution for further HPLC-FLD analysis. Fig. 1 shows the chromatograms of a spiked muscle sample extracted with the LLE and ED procedures. It is clear that the use of the ED extraction significantly reduced matrix interference with the samples.

**Fig. 2:**
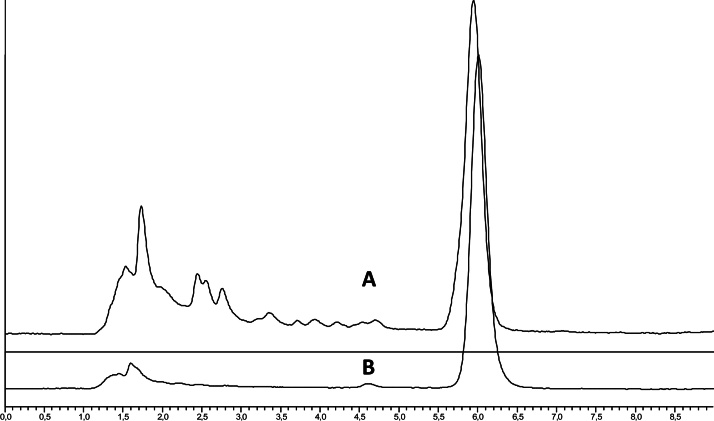
Chromatograms of a naturally-contaminated pig muscle sample extracted with (A) LLE method and (B) ED method.

Using the pig muscle, liver and kidney samples spiked with 1 μg/kg of OTA, the recovery obtained with the ED extraction method was higher and less variable than the recovery obtained with the conventional LLE sample pretreatment (Table 2). The LOD and LOQ of the ED method were also lower than the LOD and LOQ of the LLE method (Fig. 2).

## Validation

The HPLC-FLD method was validated according to Ref. [12] by evaluating: specificity, recovery, trueness, decision limit (CCα), detection capability (CCβ) of the method selectivity, linearity, LOD and LOQ, repeatability and reproducibility.

A limit of 1 μg/kg (1 ppb) OTA in pork meat and derived products was established by the Italian Ministry of Health in 1999 [13]. The validation procedure was performed taking into account the value of 1 μg/kg OTA.

Calibration curves were based on the analysis of triplicate standard solutions at six concentration levels in matrix. Liver, kidney and muscle samples spiked with OTA at 0.1, 0.5, 1, 2.5 and 5 μg/kg were analyzed using the ED and HPLC-FLD method. The experiment was repeated three times. Taking into account the concentration steps, spiked samples corresponded to OTA standard concentrations of 0.25, 1.25, 2.5, 6.25 and 12.5 ng/ml. Linear regression analysis was used to calculate the equation for the line that best fitted the calibration data and showed correlation coefficient greater than 0.995.

The repeatability was tested by analyzing liver, kidney and muscle samples spiked with OTA. Samples were spiked at the levels of 0.1 ng/g (corresponding to 2.5 ng/ml), 1 ng/g (corresponding to 2.5 ng/ml), and 5 ng/g (corresponding to 12.5 ng/ml). All samples were measured in triplicate on the same day. For the within-laboratory reproducibility test, each of the contamination levels was tested in triplicate over a period of five days. The results of these experiments were also used for the determination of the recovery. No certified reference material was available for the trueness assessment of OTA analysis in pig tissues samples. Repeatability and reproducibility data corrected with the mean recovery were used for trueness determination; trueness (%) was calculated as the mean (recovery corrected) concentration of added known amount × 100/added amount. Selectivity studies were expressed as the ability to unequivocally assess OTA in the presence of components that are expected to be present. This was evaluated by a comparison of free-OTA vs spiked samples. The LOD and LOQ were determined by the signal-to-noise approach, defined at levels resulting in signal-to-noise ratios of 3 and 10, respectively. The analytical response and the chromatographic noise were measured from the chromatogram of a blank sample extract (1 ml) to which an OTA solution was added.

The decision limit was estimated by spiking 10 muscle, liver and kidney samples at the current limit taken as the reference value (1 μg/kg). The concentration at this limit plus 1.64 times the corresponding standard deviation equals the decision limit (α = 5%). Decision capability was estimated by spiking 10 muscle, liver and kidney samples at the corresponding CCα level. The value of the decision limit plus 1.64 times the corresponding standard deviation equals the decision capability (β = 5%).

Results of the validation study are reported in Table 3. The average recoveries were between 80.9% and 106.30% with satisfactory RSD, thus fulfilling completely the performance criteria fixed by [14], i.e. recovery in the range of 50–120% and 70–110% for levels <1 and between 1 and 10 μg/kg, respectively.

## Application of the ED method to real samples

The optimized ED method was applied to pig muscle, liver and kidney samples of 5 animals obtained from local slaughterhouses. Samples were frozen at −20 °C until analysis. All samples analysed were contaminated with different amounts of OTA, as reported in Table 4.

Our ED method simulates part of the digestion process. The OTA released and then quantified was therefore probably closer to the amount really available for in vivo absorption.

Furthermore, the ED method does not use chlorinated solvents, thus providing a considerable environmental advantage.

## Figures and Tables

**Table 1:** Optimization of ED parameters.

Pancreatin solution volume (ml)	Incubation time (h)	Muscle Recovery (% ± SD)	Liver Recovery (% ± SD)	Kidney Recovery (% ± SD)
20	3	76.15 ± 0.30	80.15 ± 0.20	82.00 ± 0.10
	2	74.10 ± 0.24	82.00 ± 0.22	80.97 ± 0.20
	1	78.02 ± 0.25	83.67 ± 0.33	85.60 ± 0.12

10	3	75.65 ± 0.29	81.65 ± 0.17	80.76 ± 0.09
	2	77.54 ± 0.34	81.32 ± 0.10	81.87 ± 0.07
	1	78.65 ± 0.16	82.67 ± 0.10	83.58 ± 0.14
	
5	3	84.01 ± 0.06	82.82 ± 0.01	82.54 ± 0.03
	2	80.04 ± 0.20	82.98 ± 0.03	97.02 ± 0.03
	1	90.32 ± 0.02	92.17 ± 0.03	95.14 ± 0.04

**Table 2:** Recovery (3 replicates) ± SD, LOD and LOQ of muscle, liver and kidney samples spiked with OTA 1 μg/kg and extracted with the LLE and ED method.

Method	Muscle	Liver	Kidney
	Recovery (%)	Recovery (%)	Recovery (%)
LLE	79.90 ± 1.80	89.90 ± 0.88	90.10 ± 0.50
ED	90.32 ± 0.02	92.17 ± 0.03	95.14 ± 0.04

Method	LOD (μg/kg)	LOQ (μg/kg)	LOD (μg/kg)	LOQ (μg/kg)	LOD (μg/kg)	LOQ (μg/kg)
LLE	0.012	0.025	0.012	0.025	0.012	0.025
ED	0.002	0.001	0.002	0.001	0.002	0.001

**Table 3:** Validation parameters of ED method coupled with HPLC-FLD according to Ref. [12].

Parameters		Muscle	Liver	Kidney
LOD (μg/kg)		0.001	0.001	0.001
LOQ (μg/kg)		0.002	0.002	0.002
r^2^		0.999	0.995	0.999

Repeatability				
0.1	Mean concentration ± SD	0.082 ± 0.001	0.092 ± 0.005	0.082 ± 0.006
	RSD (%)	1.89	6.07	0.75
	Trueness	82	92	82
1.0	Mean concentration ± SD	0.80 ±0.01	0.94 ± 0.01	0.82 ± 0.02
	RSD (%)	1.83	1.05	2.53
	Trueness	80	94	82
5.0	Mean concentration ± SD	4.74 ± 0.10	5.11 ± 0.20	4.88 ± 0.14
	RSD (%)	1.37	3.94	2.77
	Trueness	95	102	98

Reproducibility				
0.1	Mean concentration ± SD	0.080 ± 0.001	0.092 ± 0.004	0.080 ± 0.002
	RSD (%)	1.96	4.53	2.82
	Trueness	80	92	80
1.0	Mean concentration ± SD	0.81 ± 0.01	0.90 ± 0.04	0.82 ± 0.03
	RSD (%)	1.61	4.20	3.31
	Trueness	81	90	82
5.0	Mean concentration ± SD	4.72 ± 0.06	4.97 ± 0.03	4.92 ± 0.10
	RSD (%)	1.22	0.58	2.12
	Trueness	94	99	98

Recovery%				
0.1		86.9 ± 1.80	85.80 ± 1.02	80.90 ± 5.00
1.0		90.32 ± 0.02	92.17 ± 0.03	95.14 ± 0.04
5.0		96.78 ± 1.30	106.30 ± 4.03	97.44 ± 2.70

CCα		1.032	1.075	1.049
CCβ		1.064	1.150	1.099

**Table 4:** OTA concentrations (μg/kg) in naturally-contaminated pig muscle, liver and kidney samples determined with ED method coupled with HPLC-FLD.

Sample	Muscle (μg/kg)	Liver (μg/kg)	Kidney (μg/kg)
1	0.12	0.59	0.91
2	0.15	0.39	0.17
3	0.11	0.07	0.23
4	0.09	0.25	0.29
5	0.20	0.45	0.28
	0.13 ± 0.04	0.35 ± 0.20	0.37 ± 0.30
